# Breakage Assessment of Lath-Like Crystals in a Novel Laboratory-Scale Agitated Filter Bed Dryer

**DOI:** 10.1007/s11095-022-03411-x

**Published:** 2022-10-17

**Authors:** Wei Pin Goh, Kushal Sinha, Nandkishor K. Nere, Raimundo Ho, Shailendra Bordawekar, Ahmad Sheikh, Mojtaba Ghadiri

**Affiliations:** 1 School of Chemical and Process Engineering, University of Leeds, Leeds, LS2 9JT UK; 2 Process Research and Development, AbbVie Inc., 1 North Waukegan Road, North Chicago, IL 60064 USA

**Keywords:** acicular, agitated filter dryer, breakability, crystals, shear deformation

## Abstract

Agitated filter bed dryer is often the equipment of choice in the pharmaceutical industry for the isolation of potent active pharmaceutical ingredients (API) from the mother liquor and subsequent drying through intermittent agitation. The use of an impeller to promote homogeneous drying could lead to undesirable size reduction of the crystal product due to shear deformation induced by the impeller blades during agitation, potentially causing off-specification product and further downstream processing issues. An evaluation of the breakage propensity of crystals during the initial development stage is therefore critical. A new versatile scale-down agitated filter bed dryer (AFBD) has been developed for this purpose. Carbamazepine dihydrate crystals that are prone to breakage have been used as model particles. The extent of particle breakage as a function of impeller rotational speed, size of clearance between the impeller and containing walls and base, and solvent content has been evaluated. A transition of breakage behaviour is observed, where carbamazepine dihydrate crystals undergo fragmentation first along the crystallographic plane [00l]. As the crystals become smaller and more equant, the breakage pattern switches to chipping. Unbound solvent content has a strong influence on the breakage, as particles break more readily at high solvent contents. The laboratory-scale instrument developed here provides a tool for comparative assessment of the propensity of particle attrition under agitated filter bed drying conditions.

**Supplementary Information:**

The online version contains supplementary material available at 10.1007/s11095-022-03411-x.

## Introduction

Agitated filter bed drying is the unit operation of choice in the pharmaceutical industry for the isolation of potent active pharmaceutical ingredient (API). Following filtration, heat is supplied to the wet cake and an impeller is agitated intermittently to enhance the heat transfer and promote homogenous drying of the drug substance. Nitrogen gas is often applied under positive pressure to purge unbound solvent from the wet cake. The shear deformation of the powder bed, induced by the rotating impeller, could result in particle breakage, agglomeration, polymorphic changes and/or process-induced disorder of the API. This is often undesirable as it could lead to further downstream processing issues, degraded product quality, and a deviation from the product specification.

Agitated drying of API, if not done appropriately could be accompanied by particle agglomeration and/or attrition, adversely affecting product quality attributes. In some cases, the drying process could cause solid-state transformations or a change of hydration, requiring controlled relative humidity environment to achieve the desired polymorphic outcome. Following the filtration stage, typically about 10–40% of solvent content is retained in the wet cake. As the particle bed dries down to the critical solvent level, agglomerates are formed due to capillary bridges [1–3]. Further drying may cause particle breakage to become more dominant as a result of increased frictional contacts of particles. Attrition and agglomeration can occur simultaneously during drying. There are many process parameters that could alter the behaviour of the particle bed in terms of particle breakage and agglomeration. Bagster and Bridgwater [4] conducted a study on material flow in an agitated dryer and found that the dynamics of the bed is influenced by the blade height and angle and bed depth. Lekhal *et al*. [1, 2] have shown that the final crystal properties are affected by process parameters such as agitation rate, temperature and pressure. They also showed that low drying rate and/or high shear rate tends to promote particle breakage, while agglomeration is dominant at high drying rate and/or low shear rate. Impact breakage is present only at high impeller speeds. Particle breakage as a result of shearing of the particle bed is more pronounced and dominant [5]. To study particle breakage due to bulk shear deformation, Paramanathan and Bridgwater [6] developed a special shear cell, where attrition in a defined failure zone under controlled stress, shear strain and strain rate could be analysed.

Agitated drying is often operated at an elevated temperature to speed up the drying rate. Material properties such as hardness and Young’s modulus are temperature-dependent [7]. Hassanpour *et al*. [8], using *α*-lactose monohydrate and Olusanmi *et al*. [9], using aspirin, have shown that the extent of breakage of these crystals increases as the temperature is increased, as the mechanical properties accountable for particle strength, i.e. hardness, stiffness and fracture toughness are strongly dependent on temperature. They used the impact breakage model of Ghadiri and Zhang [10] and analysed the breakage data in terms of the functional group *H/K*_*c*_^2^, representing the material properties, where *H* is hardness and *K*_*c*_ is fracture toughness, decreases with temperature.

**Fig. 1:**
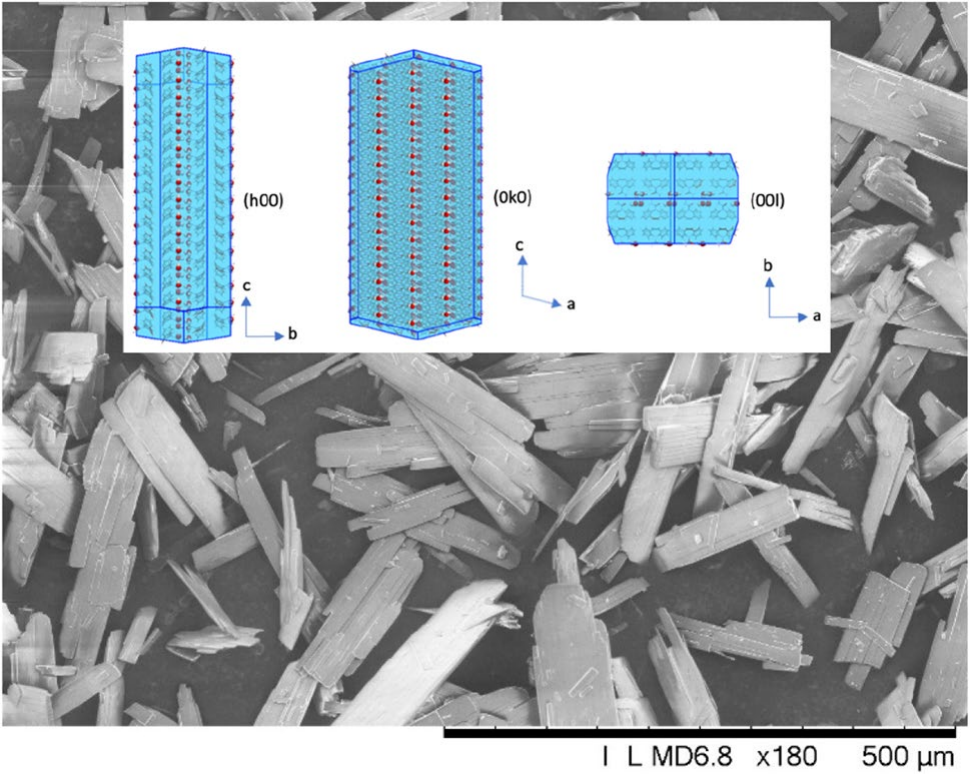
Carbamazepine dihydrate crystals viewed by scanning electron microscope (SEM) and their crystallography arrangement (generated by Mercury software, CCDC, https://ccdc.cam.ac.uk/Community/csd-community/FreeMercury/).

Development of an optimal drying process in an AFBD requires an assessment of the influence of the drying kinetics and agitation intensity on the physical/chemical attributes and stability of the compound. Over the years, many different laboratory-scale agitated dryers [11–14] have been developed to investigate the agitated drying process, but most of them are in-house instruments, developed in academia and hence not readily accessible to the pharmaceutical industry. Custom laboratory setups that mimic heat and mass transfer characteristics and applied mechanical forces at large-scale operations require a substantial quantity of materials that are unavailable at the early process development stage. Moreover, they also lack the flexibility for customisation, i.e. interchangeable blade design and precise impeller motion control. From an industrial perspective, the lack of standardized laboratory equipment for scale-down experimentation that accurately mimics the large-scale conditions has been identified as a major technology gap [15]. To address this issue, a laboratory-scale AFBD has been developed in collaboration with Freeman Technology, as an auxiliary accessory for use with the FT4 Powder Rheometer®. In this paper, we present its development and its application to the breakage behaviour of carbamazepine dihydrate (hereinafter referred to CBZ.2H_2_O) as a model API. When crystallizing in alcohol-water mixtures, CBZ.2H_2_O crystals are thin, platy and long, as shown in Fig. 1, and frangible, making them highly prone to attrition. Our previous studies have shown that CBZ.2H_2_O is susceptible to breakage due to crystal mechanical anisotropy, internal stresses developed from drying and/or defects influenced by the crystallisation conditions [16]. Here, several key AFBD process parameters are analysed, such as the impeller clearance, rotational speed, number of impeller revolutions, and solvent content. The resulting breakage is quantified using a new approach proposed for lath-shape crystals by assessing the degree of shifting of the size distribution.

## Key Operational Elements of Agitated Filter Bed Dryer

Agitated filter bed dryers vary in scale and could accommodate product mass of a few kg to hundreds of kg. A typical AFBD consists of 1) a jacketed vessel with a preferred material of construction, e.g. hastelloy, 2) a drive assembly with a heated agitator system capable of rotational and axial movement, 3) heated filter plate with interchangeable filter media, 4) dust filter, and 5) side discharge and sampling valves. During the drying process, the impeller may be rotated intermittently and also programmed to induce axial mixing by going upward and downward as per need basis. For a pilot-scale drying, the diameter of the impeller can typically be in the range of 0.08–2 m, rotated at speeds in the range of 0–25 RPM, though for smaller scale dryer, the speed could go up to 100 RPM or more. The clearance of the impeller from the base of the vessel at lowermost position is dictated by the size of the equipment. Similarly, a clearance exists between the impeller blade tip and the vessel wall. The operating volume of wet cake is dictated by the uppermost and lowermost position of the impeller. However, to ensure the efficiency of the drying process, normally a bed height of two to three times the impeller blade height is used.

### Filtration and Washing

Right after the completion of crystallisation, the slurry is transferred into the AFBD to remove the mother liquor from the crystallised product. A number of washes, either in a re-slurrying or displacement mode, are applied where appropriate to flush out the impurities using wash solvent that has similar composition to the mother liquor. In some cases, wash solvent having different composition could also be used when it proves beneficial to do so, e.g. reducing agglomeration, better purging of impurities, etc. Once the mother liquor is filtered and the wet cake settled, vacuum or headspace pressure can then be applied to deliquor the excess unbound wash solvent. Blowdown of the wet cake using nitrogen gas to drive the solvent out could also be done if necessary. During the deliquoring and blowdown process, smoothing of the wet cake using the impeller may be performed (typically 3–5 impeller rotations) to fill-in any cracks formed on the free surface, avoiding the bypass of nitrogen flow to increase the efficiency of solvent removal.

### Drying with Heating

Blowdown and deliquoring will not remove all the solvent in the wet cake. Very often, the wet cake is heated up to the upper temperature dictated by the thermal stability of the API to facilitate the drying process. The heating rate is governed by the loss on drying (LOD) of the solvent content and propensity of hardening of the wet cake (very likely to happen when the drying rate is fast), which is mostly dependent of the extent of solvent bridging and resulting solid bridge formation. Vapour removal can be accelerated by applying nitrogen gas to the system either through headspace purging or through the wet cake. Vacuum can also be applied from the top or bottom based on the type of dryer employed. In certain cases, drying is done under controlled relative humidity environment and temperature to achieve the desired crystal form.

### Intermittent Agitation

Upon reaching the desired drying temperature and LOD, the impeller is then lowered to agitate the partially-dried cake intermittently to accelerate the drying. The typical speed used in industrial operation is approximately 5 to 10 RPM. For materials that are prone to attrition, a rotational speed as low as 2 RPM is used. For a typical dryer with diameter of 2 m, the maximum tip speed is around 2 m/s. Heat supply through the impeller is very common in most of the filter bed dryers to enhance the drying rate of the partially-dried cake. Some dryers also allow heating from the bottom plate. The intermittent mixing of the partially-dried cake is continued until an acceptable solvent content is achieved, according to International Conference on Harmonization (ICH) Standards or product specification targets. The typical drying time can range from hours to days, depending on the API physical properties to be controlled, crystal form and the type of solvent used [17, 18].

## Development of a Laboratory-Scale Agitated Filter Bed Dryer

Use is made of the FT4 Powder Rheometer (Freeman Technology, Tewkesbury, UK) as a chassis for the AFBD and to provide control of the impeller, due to the ability to precisely regulate rotational velocity and accurately measure torque, taking account of the following requirements:

1. Interchangeable impeller blade geometry to assess the efficiency of blade design in promoting homogeneous drying
2. Variable impeller rotational speed
3. Programmable impeller motion for devising optimal protocol for drying
4. Heating capability for assessment of drying at elevated temperature
5. Filtration capability with high chemical resistance
6. Vacuum capability for isolation of product from waste liquor
7. Array of sensors probing the temperature, pressure and humidity at various stages during agitated filter bed drying

**Fig. 2:**
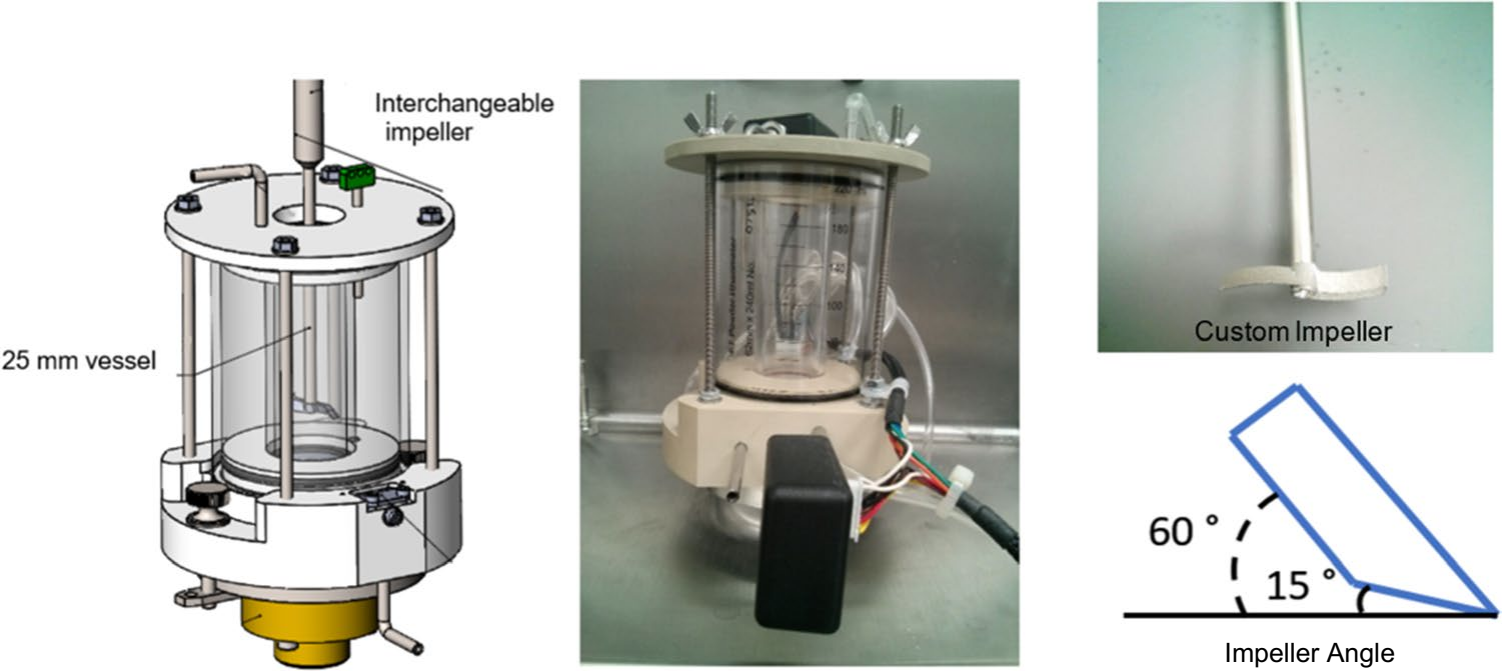
Schematic of the AFBD unit (left), the image of the actual prototype (middle), and the scale-down conventional AFBD impeller blade by Selective Laser Sintering (right).

A perspective drawing and a picture of the actual prototype of the AFBD unit are shown in Fig. 2 (left and middle). It is jacketed so that hot fluid can be circulated through the vessel to heat up the wet cake. The inner vessel is 25 mm in diameter, and it is equipped with an interchangeable impeller. The impeller is a geometrically scaled down replica (24.5 mm in diameter) of a conventional AFBD impeller, adopted from Hare’s work [19]. It was 3D-printed by selective laser sintering as the unique curvature of the blade, important for agitation, is difficult to be fabricated through traditional CNC machining. The blade has an inclination of 60° to the horizontal and the edge of the blade is chamfered to 15° as shown in Fig. 2 (right). The functions of the impeller are directional, in a sense that rotating the impeller in clockwise direction impose ‘spreading’ to the powder bed to effect smoothing, while in anti-clockwise direction, the impeller is conditioning the powder bed by cutting and lifting the bed to promote mixing.

**Fig. 3:**
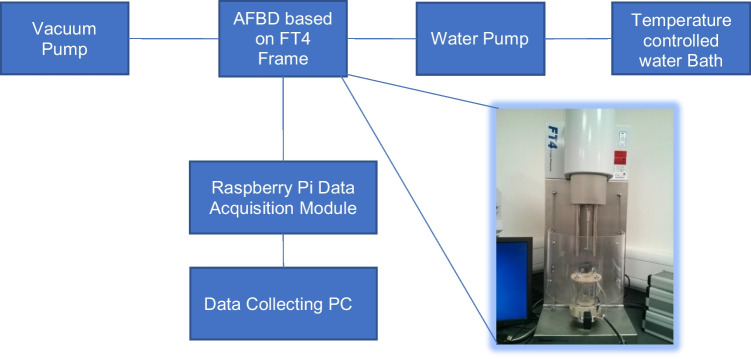
System overview of the new AFBD device.

To fully realise the features listed above, several auxiliary units are added, as listed in Fig. 3. The filtration capability is provided by having a porous base and attaching a vacuum pump to it. To be able to circulate hot fluid through the jacketed vessel, a water pump is used to draw the hot fluid from the temperature-controlled water bath back and forth to the AFBD unit. A Raspberry Pi data acquisition module is used to receive real-time data from the array of sensors attached to the AFBD unit. Collection of the data is performed by a PC through wireless ad-hoc network. The graphical user interface of the data acquisition software and the list of the parameters that the AFBD unit could monitor are given in Fig. S1 (Supplementary Information).

## Materials and Methods

### Carbamazepine Dihydrate

The API crystals used in this work are carbamazepine dihydrate (C_15_H_16_N_2_O_3_), a drug primarily used as an anticonvulsant to treat epilepsy and neuropathic pain [20]. The crystals were prepared by addition of carbamazepine-ethanol solution to antisolvent (water) and dried at room temperature for two days. There are two water molecules embedded in the crystal lattice structure, forming channels of water molecules along the largest dimension of the macroscopic crystal, parallel to the crystal axis *c* (refer to Fig. 1). Its structure is monoclinic with a space group *P*2_1_/*c* [21]. It is highly platy and long, brought about by differences of the growth rate of the faces; those of (h00) and (0k0) of the crystals are substantially lower than the third dimension (Fig. 1). Crystal axes *b* and *c* correspond to the particle width and length of the grown crystals. The (0k0) planes, which are perpendicular to the dominant face (h00), are key to the mechanical properties of carbamazepine dihydrate, as they represent the main cleavage planes within the crystal structure. These planes are weak as molecular layers formed by π–π stacking can delaminate across the (0k0) surface. In addition, waster channels also run along this plane. Previous work have shown that crack formation across the (0k0) planes could be caused by release of mechanical strain from the effect of mechanical impact, or breakage of the carbamazepine dimers as a result of dehydration [16, 22]. Bending force caused by the elongated morphology of CBZ.2H_2_O crystals would also exert breakages perpendicular to the longest crystal dimension, i.e. the (00l) planes [16, 23].

### Subjecting Carbamazepine Dihydrate Crystals to Different Process Treatments

Initially, 1 g of dry sample was fed into the AFBD vessel, which filled up the vessel to about three times the impeller blade height. The impeller was then lowered down to 20 mm from the base before rotating it at 10 RPM in anti-clockwise direction to promote mixing of the powder bed, while still lowering it until it reached the designated clearance. The impeller was then rotated in the same direction at fixed height to simulate the agitation process in an AFBD. Several process parameters, such as impeller tip speed, agitation duration, solvent content, extent of cake consolidation which are key design elements of AFBD process for physical properties control, were tested, and the detailed conditions used are discussed in the results and discussions section accordingly.

### Particle Size Analysis

After each process treatment, the crystals were collected, and their size distributions were measured using Morphologi G3 of Malvern Panalytical using dry dispersion technique, and expressed on a volumetric basis. The lowest allowable dispersion pressure (0.5 barg) was used to disperse the sample. The analysis procedure for particle size follows the approach of Goh *et al*. [24], as briefly described below. It was determined that 0.5 barg dispersion pressure pulse does not cause any observable particle breakage, as compared to the wet dispersion analysis. Due to the fact that the crystal thickness is significantly smaller than the width and length and that on breaking, the thickness does not change, the area made up by the latter two is assumed to be representative of the volume of the crystal. Therefore, the projected area of the crystals is measured and the size distribution curves are produced. The extent of particle breakage, denoted by R*, is defined as the degree of shifting of the size distribution curve, i.e. the positive difference of area under the curve by subtracting the feed PSD from the PSD of sample subjected to breakage. The more the PSD shifts away (to the left) from the reference PSD, the higher the extent of particle breakage. This can be calculated using the equations below:

1 $${S}_i={B}_i-{B}_{ref,i}$$

2 $${R}^{\ast }=\sum\nolimits_{i=1}^n{S}_i\left[{S}_i>0\right]$$

where *S*_*i*_ is the difference between the area percentage of the reference size distribution curve, *B*_*ref,i*_ and the size distribution of broken particles, *B*_*i*_ in the bin number *i* and R* the sum of the positive differences between the two size distribution curves up to bin number *n*.

## Experimental Results and Discussions

### Size Distribution of Feed Material

**Fig. 4:**
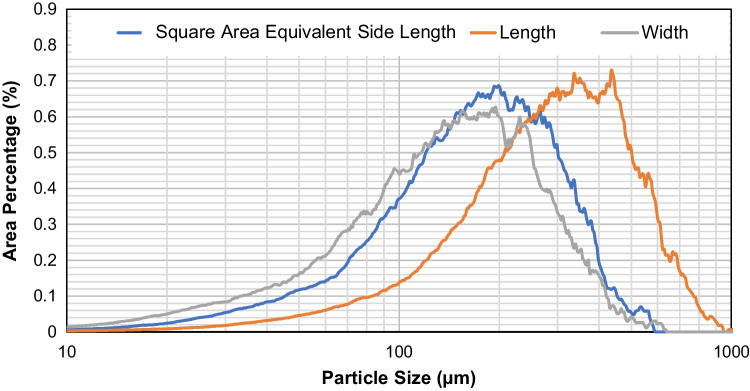
Distributions of the square-area-equivalent side length, length and width of feed carbamazepine dihydrate crystals.

The size distribution of the feed sample is shown in Fig. 4. Three different sizes are presented here, namely the square-area-equivalent side length, actual length and width of the crystals. The square-area-equivalent side length is defined as the side length of a square that has the same area as the projected shadow of the crystal captured using Morphologi G3. The mode of the length is approximately 350 μm, while the mode for the width is approximately 200 μm. The characteristic sizes of the distributions are shown in Table I**.**

**Table I:** D-Values of the Square-Area-Equivalent Side Length, Length and Width of Feed Carbamazepine Dihydrate Crystals

	Square-area-Equivalent Side Length (μm)	Length (μm)	Width (μm)
*D*_10,3_	62	109.8	44.8
*D*_50,3_	168.7	287.9	140.9
*D*_90,3_	325.1	539.2	290.4

### Effect of Impeller-Base Clearance

Shear deformation of the crystals is most prominent at the region between the impeller blade and the vessel base, where the shear band prevails and the clearance is highly influential on the stresses experienced by the particles within this gap. Too small the gap, the particles could undergo extensive breakage due to crushing. Three different clearances of 1, 3 and 5 mm were tested in this work. The time and speed of the impeller rotation are 40 minutes and 120 RPM, respectively. The ratio of particle diameter to clearance size is difficult to be quantified as the particles used had a full-size distribution, instead of a narrow size range. To simplify this, the mode of the length distribution of the feed crystals is used as a reference, owing to the high tendency of CBZ.2H_2_O to break perpendicular to the length. The particle to clearance ratio is then worked out to be 2.8, 8.5 and 14.3 particle length for clearance sizes of 1, 3 and 5 mm, respectively. Interestingly, the change of the clearance size has only minor influence on the particle size distribution. The extent of breakage obviously increases as the gap is made smaller, increasing from ~5% for 5 mm gap size to ~9% for 1 mm gap size. The smaller the clearance size, the larger the extent of breakage. The difference in R* between the 3 and 5 mm clearance sizes is very small, compared to that of 1 mm, indicating that the particle breakage of both of these cases are due to the shear deformation of the powder bed induced by the impeller and not crushing. The evolution of the particle size distribution is presented in terms of their characteristic sizes, *D*_10,3_, *D*_50,3_ and *D*_90,3_ in Supplementary Information, Fig. S2. The smaller the clearance size, the smaller the characteristic sizes of the treated samples. Selecting an appropriate clearance is critical in minimising unwanted breakage of particles while promoting a homogeneous drying across the powder bed during agitated drying process.

### Effect of Impeller Speed

**Fig. 5:**
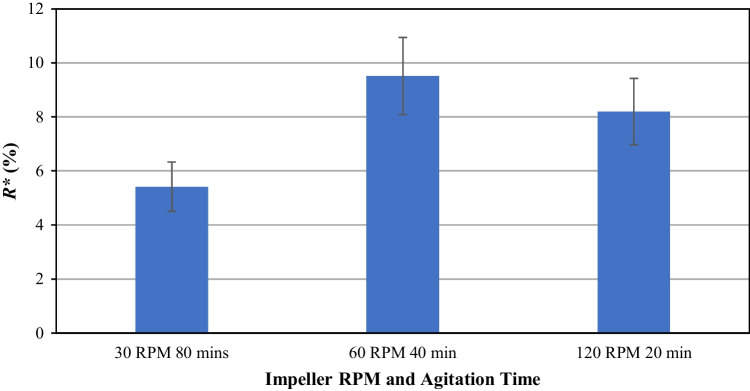
R* of the broken carbamazepine dihydrate crystals as a function of impeller speed at 1 mm clearance, keeping the number of rotations constant.

Three different impeller rotational speeds of 30, 60 and 120 RPM were used to agitate the particle bed, corresponding to the impeller tip speeds of 0.04, 0.08, 0.15 m/s (Fig. 5), while keeping the number of rotations constant. These are lower than the typical tip speed used in an industrial AFB drying operation, but nevertheless provide a comparative study of the sensitivity of the model crystal breakage to strain rate. It was found that for the range of speeds used here, the extent of breakage of CBZ.2H_2_O crystals was not sensitive to the impeller speed. Increasing the impeller rotational speed by four times increases the R* by only 3%. Agitation at 60 RPM causes the highest breakage extent of CBZ.2H_2_O, at ~9.5%, but when compared to the other two impeller speeds tested, the difference is not significant. The PSDs of the samples agitated are compared against the feed to assess the role of impeller tip speed in the breakage of CBZ.2H_2_O crystals (Supplementary Information, Fig. S3). The characteristic D-values of the distributions are shown in Fig. S4 (Supplementary Information). A gradual decrease in the *D*_90,3_ is observed as the impeller tip speed is increased. However, the *D*_10,3_ and *D*_50,3_ of the distributions do not show such trend. Depending on the nature of the materials, some powders are highly sensitive to strain rate and could result in drastic shift of their particle size distribution. The ability of the current AFBD setup to study the powder behaviour under different strain rates is highly desirable, providing useful information for developing drying protocols that are best suited for different powders of interest.

### Effect of Number of Impeller Revolutions

**Fig. 6:**
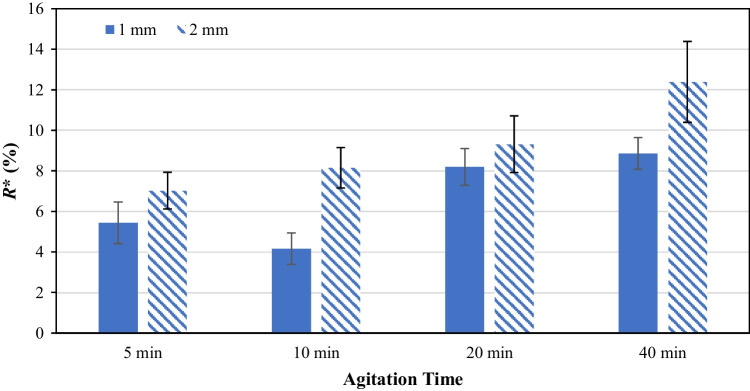
R* of the broken carbamazepine dihydrate crystals as a function of agitation time at 120 RPM at 1 mm and 2 mm clearance.

Carbamazepine dihydrate crystals were subjected to four different lengths of agitation time (5, 10, 15 and 20 minutes) while keeping the rotational speed constant. The extent of particle breakage calculated as a function of agitation time is shown in Fig. 6, corresponding to 600, 1200, 2400 and 4800 number of revolutions. Increasing the agitation time does increase the particle breakage to a certain degree, but that is not always the case. At 10 minutes agitation, the calculated R* is slightly less compared to that of 5 minutes, but this is within the span of experimental error as shown by the error bar for three number of repeats. A clear difference is seen when the agitation time is increased to 20 minutes, but further increase in the agitation time to 40 minutes yields no further breakage of the crystals. This is likely due to the fact that as the crystals reduce in size, they become harder to break. Also, the fines generated help facilitate sliding, commonly referred to as three-body rolling, and hence reduce the interlocking between the particles and the frictional contact force between them. The resulting PSDs of the crystals agitated for different lengths of time are shown in Fig. S5 (Supplementary Information) and the characteristic D-values are shown in Fig. S6 (Supplementary Information). Interestingly, when similar operating conditions were used but with 2 mm clearance instead of 1 mm, the breakage of CBZ.2H_2_O crystal followed the trend hypothesized, which stipulated that the extent of particle breakage increases with the agitation time. A gradual increase of the R* is found when the agitation time is increased. It is also worth noting that the extent of breakage of the samples agitated at 2 mm clearance is generally higher than those samples agitated at 1 mm clearance. The exact reason is unclear and requires further studies of transient jamming leading to particle breakage, implying that 1 mm clearance is too small for particles to get nipped.

### Effect of Vacuum Suction (Cake Consolidation)

**Fig. 7:**
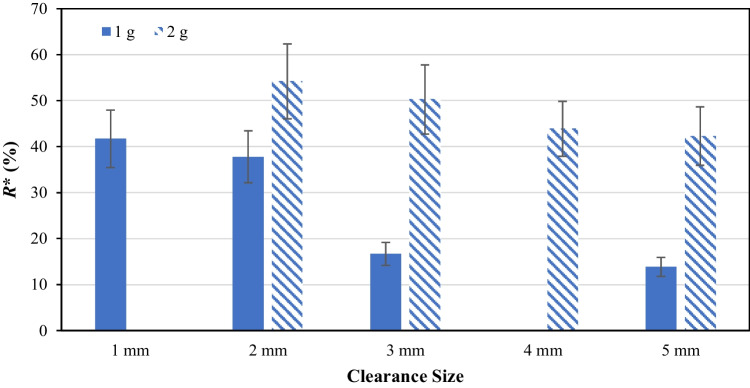
R* of the broken carbamazepine dihydrate crystals as a function of clearance size at 120 RPM with vacuum suction applied.

In industrial operations, during the drying stage, the system is closed and the application of vacuum to the vessel generally does not cause increased compression on the bed. However, the wetcake could be compressed and consolidated due to its own weight, as well as by pressure differential across the bed during the wash cycles. Because of the smaller scale, the stresses experienced in laboratory-scale setup are significantly lower than the corresponding industrial case. In order to artificially increase the compressive stresses to simulate industrial conditions, vacuum suction can be applied. The increased compression stress and corresponding shear stress when agitating enhances attrition. In this work, a vacuum pump was connected to the bottom of the AFBD vessel to investigate the effect of vacuum suction application on the breakage of CBZ.2H_2_O crystals. Depending on the particle size and bed packing, the pressure drop across the powder bed varied from 50 to 250 mbar. Applying vacuum suction to the bottom of the AFBD vessel imposes additional normal load to the powder bed due to the pressure drop as the air percolates through the pack bed. The extent of particle breakage as a function of impeller-base clearance with vacuum suction applied are shown in Fig. 7. The first thing that can be noticed is that the extent of particle breakage is much higher compared to the same set of experiments performed without vacuum suction. At the clearance size of 1 mm, the resulting R* is as high as 41%. The extent of breakage is reduced as the clearance size is increased. Going from 2 to 3 mm, a large reduction in R* of about 20% is observed. The R* at 3 and 5 mm clearance sizes is ~17 and 14%, respectively. This indicates that there may exist a critical clearance size somewhere between 2 and 3 mm, below which the particle interlocking causes extensive breakage. Above the critical clearance size, the effect of clearance size diminishes and R* does not vary much with further increase in the clearance size. Another set of experiments were performed, but with 2 g of sample instead of 1 g. The R* calculated for the new set of experiments is shown in the same figure, respectively. The large drop in R* observed earlier is not seen in the new set of data. Comparing the difference between 2 and 3 mm clearance sizes, the decrease in R* is gradual and reasonable, unlike the previous dataset. Having compared the results from these datasets, it can be deduced that the sudden drop of R* in the first dataset is likely due to insufficient sample present above the impeller blades. In the second data set, where the sample mass is doubled, R* is reduced at a steady rate as the impeller-base clearance is increased. The extent of breakage is high even for the largest clearance size (5 mm) tested, achieving a value of ~42%. This indicates that CBZ.2H_2_O crystals are indeed very weak and prone to breakage.

The combined effect of impeller rotational speed and application of suction was also investigated, but not shown here for brevity. Interestingly, the trend observed for the samples with suction is the same as those without. The R* increases when the impeller rotational speed is increased from 30 to 60 RPM, but reduces when it is increased to 120 RPM. Similar trend is also observed for the samples agitated at a larger clearance size of 3 mm with vacuum suction applied. When the impeller is rotating, the particles in front of the blades are lifted up and fall over to the back of the blades. The speed of those particle being lifted up by the impeller blade to fill the void behind the blades is dictated by the gravity and any additional normal load exerted on the powder bed, i.e. vacuum suction (typically up to 25 kPa). Increasing the impeller rotational speed does increase the extent of particle breakage for CBZ.2H_2_O, but only when the dynamics of the powder bed stays the same. At high rotational speed, the powder bed is in a dilated state, as the particles are lifted up faster. In some extreme cases, the powder bed could get fluidised, but that is not the case here due to the vacuum suction applied. The consistent trend shown by the three different datasets presented suggests that there is indeed a change of powder bed dynamics to a more dilated state when the impeller speed is increased from 60 to 120 RPM, hence causing less particle breakage.

The R* of CBZ.2H_2_O crystals agitated at different periods of time with vacuum suction applied is also investigated. Again, applying vacuum suction incurs significant increase in the R*. It is found that increasing the agitation time increases the extent of breakage of CBZ.2H_2_O. However, the data trend seems to reach a plateau when the particles are being agitated for 20 minutes, as a further increase in the agitation time results in no significant increase in R*. This trend requires further investigation, as particle breakage under bulk shear deformation is a highly complex process due to particle size and shape changes. This will result into a change in particle bed rheology, altering the contact force distribution. However, the analysis of this process is outside the scope of this paper and can be addressed by Discrete Element Method (DEM).

### Effect of Liquid Content

**Fig. 8:**
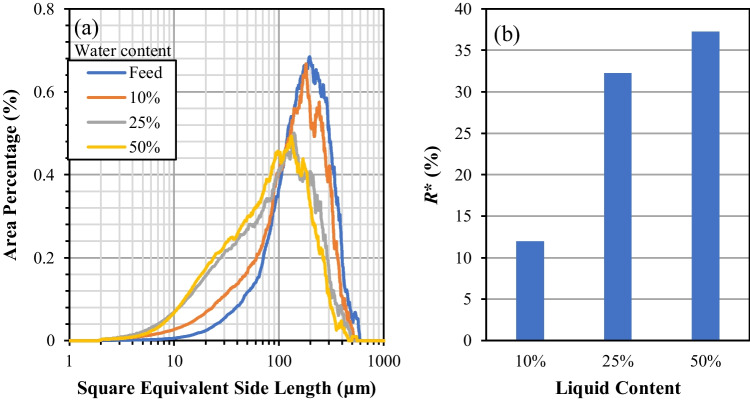
PSDs (**a**) and R* (**b**) of the broken carbamazepine dihydrate crystals as a function of liquid content.

The presence of liquid between the particles could influence the resistance to flow of the powder bed, and that is governed by the type and amount of liquid present in the powder bed. CBZ.2H_2_O crystals in this work were crystallised using a water-ethanol solution. Water acts as anti-solvent to the crystallisation process, due to very low solubility of CBZ.2H_2_O in it, ~311.1 μg/ml [25]. To study the effect of liquid content present in the powder bed on the breakage behaviour of CBZ.2H_2_O, an aqueous slurry was first prepared. 2 g of CBZ.2H_2_O crystals were mixed with 10 ml of water and stirred gently using a spatula to wet the crystals thoroughly. The slurry was then filtered in the AFBD vessel until a desirable solvent content was reached. The reduction of liquid content by mass was monitored using the FT4 normal load sensor. The wet cakes with different liquid contents were then agitated at 120 RPM with an impeller-base clearance of 2 mm to assess their role in the breakage of CBZ.2H_2_O. No vacuum suction was applied. Short intermittent agitation time (5 s) at 120 RPM was used, as the wet crystals had a tendency to get suspended on top of the impeller blade due to the liquid bridges formed between the particles having higher attraction force compared to the gravitational pull. Hence, after every agitation step, the impeller blade was lifted up to 20 mm, well above the sample, and lowered back down to mix the powder bed. The total agitation time was 5 minutes and after that the sample was left in the AFBD vessel with vacuum suction applied to be air dried at room temperature for 2 hours. The dried samples were then collected for further analysis using Morphologi G3. Three liquid contents of 10, 25 and 50% of water by mass were tested, i.e. used at the start of shearing the bed by the impeller. The PSDs of the CBZ.2H_2_O samples agitated at different liquid contents are shown in Fig. 8 (left). It is evident that solvent content is influential on the reduction of particle size. The higher the liquid content in the powder bed when it is being agitated, the higher the extent of breakage of the crystals. The R* calculated for each liquid content is shown in Fig. 8 (right). At 10% liquid content, the R* is about ~12% and it increases to ~32% and 37% when the amount of water in the powder bed is increased, corresponding to the liquid contents of 25% and 50%, respectively. This is expected as a decrease in liquid content in the powder bed reduces the attraction force between the particles, which in turn leads to less resistance of the powder bed to flow when agitated by the impeller. Unbound solvent can also induce particle agglomeration within the critical liquid content range [3]. However, for CBZ.2H_2_O, the net effect of liquid content on particles breakages is more pronounced than that on agglomeration. These results have shown that the optimal time for applying agitation is when the cake is sufficiently dry. Applying agitation when the cake is still wet can cause severe breakage of the particles and should be avoided. It is also worth noting that beyond a critical point, at high liquid content, the liquid present could act as a lubricant and reduce the bulk friction of the powder bed.

### Shape Analysis of the Broken CBZ.2H_2_O

**Fig. 9:**
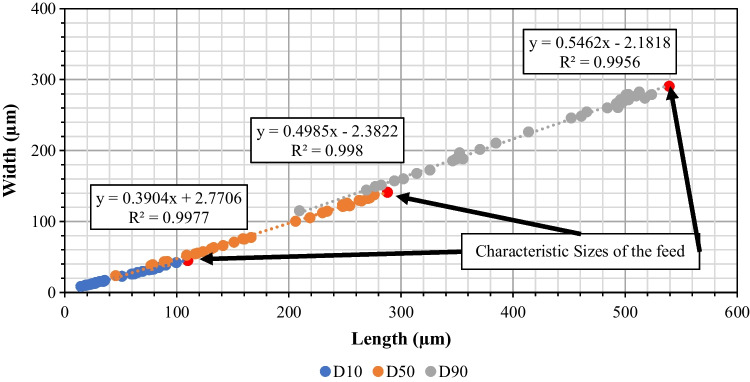
Scatter plot of the characteristic D-values of width and length distribution of carbamazepine dihydrate crystals.

**Fig. 10:**
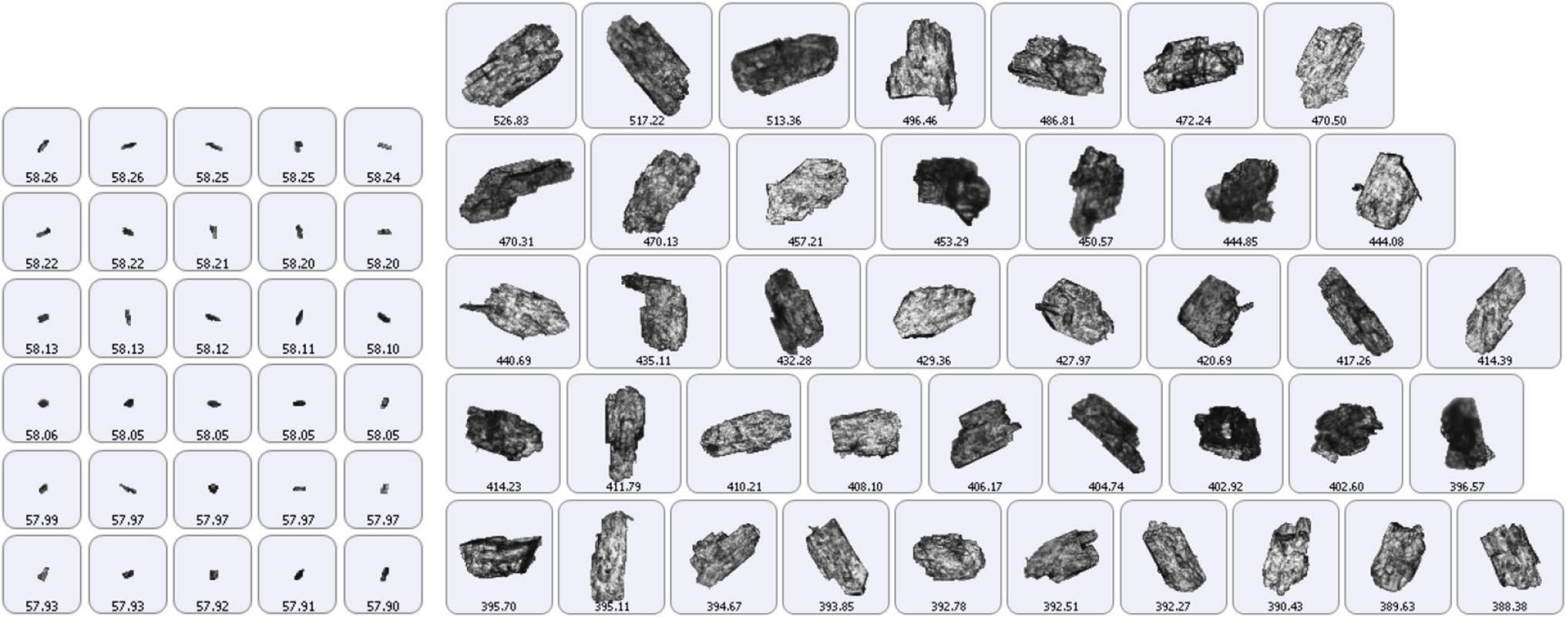
Light micrographs of small (left) and large (right) particles (treated sample).

The change in the crystal shape due to breakage under shear deformation in the AFBD was characterised by image analysis. The reduction in width and length, expressed in their characteristic particle sizes at 10, 50 and 90 cumulative volume percentages (D-values) is shown in Fig. 9. Three series of data points are given: the blue one corresponds to *D*_10,3_, orange to *D*_50,3_ and grey to *D*_90,3_ of the size distributions (length and width) of the broken particles due to different process treatments. Each data point corresponds to a set of process conditions tested in earlier section to break the crystals. The correlation between the length and width of the broken crystals subjected to different process treatments shows a strong linear relationship between the characteristic sizes of the length and width distributions. The slope of the regression line of each data series gives a measure of the average aspect ratio of the broken crystals. D_90,3_ is associated with the biggest particle group in a distribution, while D_10,3_ represents the smallest particle group. The slopes of the regression lines are found to be 0.39, 0.49 and 0.54 for D_10_, D_50_ and D_90_, respectively. There is a trend of reducing aspect ratio with particle size but the extent is not much even though the crystals have been subjected to substantial breakage. There are two breakage mechanism at play here: (i) fragmentation of the crystals, i.e. (00l) perpendicular to their largest dimension, snapping them into smaller pieces and (ii) cleaving of crystals along the crystallography planes of weakness, i.e. (0k0) which are parallel to their largest dimension. As a result of these two mechanisms happening concurrently, the change of aspect ratio is not as significant as expected. The micrographs of some of the biggest and smallest crystals of one of the agitated samples (with 10% liquid content), obtained by light microscopy, are shown in Fig. 10. It can be seen that the big crystals are generally very wide, but not so long in length. Conversely, it is very clear that those smaller crystals are slender in general, which explains the trend that was observed in Fig. 9. Evidently CBZ.2H_2_O crystals could break either by chipping off the corner or snapping along the longest dimension. This observation has also been reported by Goh *et al*. (2019) for impact breakage due to aerodynamic dispersion.

### Energy Utilisation

During agitated filter bed drying, mechanical energy is consumed to rotate the impeller. This is spent on both agitating the powder bed and causing particle attrition. The input rotational energy to the powder bed can be calculated from the torque, *T*, and angular displacement, *θ,* using the equation below:

3 $${E}_{Rotational}= T\theta$$

**Fig. 11:**
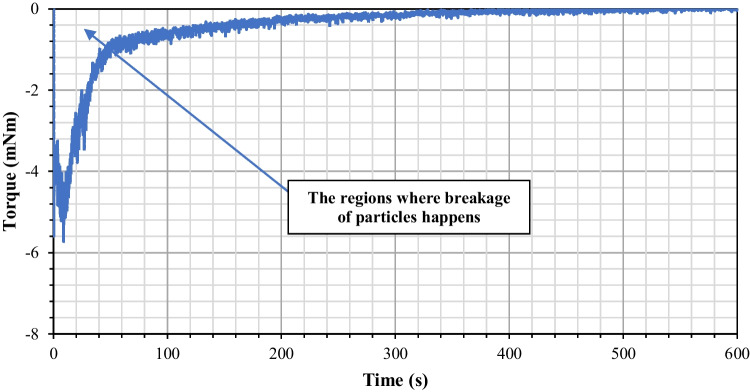
Torque profile recorded with vacuum suction applied.

**Fig. 12:**
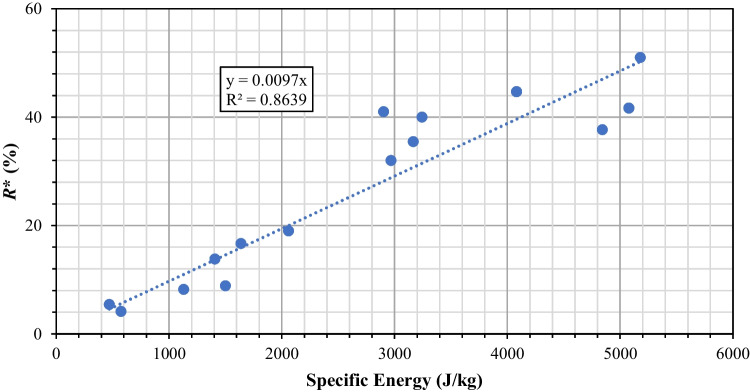
R* *vs* specific input energy.

An example of the torque profile recorded with vacuum suction applied is shown in Fig. 11. The negative sign indicates an anticlockwise rotation of the impeller blade. The magnitude of the torque increases sharply initially and then gradually before reaching a plateau. When agitation first starts, the big crystals in the powder bed are interlocked against each other. To shear the powder bed, a substantial amount of mechanical energy is needed and that explains the initial spike of the torque recorded. As the powder bed starts to flow, the shear deformation-induced on the powder bed causes the particles to break. As the particles get smaller in size, their resistance to flow becomes less, and hence a gradual decrease in the magnitude of the torque is observed. Using eq. (3), the total energy transferred to the bed can be calculated. The input mechanical energy for the experiments performed was calculated and the relationship between the R* and the specific input energy is drawn shown in Fig. 12. A reasonably good fit is obtained from linear regression, indicating that the extent of particle breakage is proportional to the expended input energy.

## Conclusions

A new test rig to simulate agitated filter bed drying has been developed in collaboration with Freeman Technology using existing FT4 Powder Rheometer. The highly versatile device performs the essential features of an industrial-scale agitated filter bed dryer, capable of filtration, programmable axial and radial mixing, temperature and humidity control, vacuum and nitrogen purge, impeller blade customisation, and process monitoring via built-in sensors for torque, temperature, pressure and humidity.

The new AFBD unit is used to assess the breakage of CBZ.2H_2_O crystals. Being long and platy, optical image processing and analysis is used to characterise the shift in particle size and shape. The extent of breakage, R*, is calculated by assessing the shift of the size distribution of the broken crystals from the feed. R* is increased when the impeller-base clearance in reduced. The impeller tip speed does not influence the breakage of CBZ.2H_2_O greatly for a given number of impeller rotations/revolutions. Increasing the number of impeller revolutions increases the extent of breakage, as intuitively expected. Applying suction to exert cake consolidation increases the extent of breakage significantly, as the resistance to shearing is increased. Liquid content is another major factor in AFBD that could cause significant particle breakage. Water was used here, and at high liquid content (50% water), the particles are held together strongly by the capillary bond between the particles. Agitating the powder bed at this stage causes significant particle breakage. When the liquid content is lowered, the breakage of the crystals is reduced proportionally as the attraction force between the particles is reduced. CBZ.2H_2_O crystals undergo fragmentation first, followed by chipping when the particles become more equant. The mechanical energy expended by the impeller correlates well with the extent of particle breakage. The proposed laboratory-scale instrument provides a tool for comparative assessment of the propensity of particle attrition under agitated filter bed drying conditions.

## Supplementary Information

ESM 1 (DOCX 592 kb)
