# Supplementary material for: Breakage Assessment of Lath-Like Crystals in a Novel Laboratory-Scale Agitated Filter Bed Dryer
Source: Pharm Res. 2022 Oct 17;39(12):3209–21. doi: 10.1007/s11095-022-03411-x (PMC9780139; doi:10.1007/s11095-022-03411-x)
Supplement: Supplementary file 1 — (DOCX 592 kb) [file 11095_2022_3411_MOESM1_ESM.docx]

**Supplementary Data**


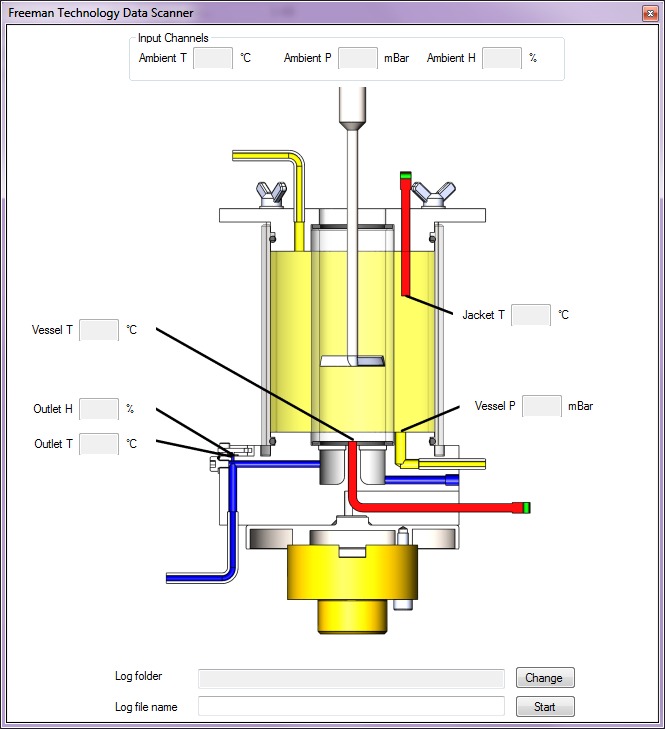


**Parameters Monitored**

- - Ambient Temperature
  - Ambient Pressure
  - Ambient RH
  - Jacket Temperature
  - Vessel Temperature
  - Outlet Temperature
  - Outlet RH
  - Outlet Pressure
  - Impeller Torque
  - Normal Force/Load

Figure S1 Software GUI and the list of parameters monitored

Figure S2 D-values of the broken carbamazepine dihydrate crystals as a function of clearance size at 120 RPM agitated for 40 mins

Figure S3 PSDs of broken carbamazepine dihydrate crystals as a function of impeller speed

Figure S4 D-values of the broken carbamazepine dihydrate crystals as a function of impeller speed

Figure S5 PSDs of the broken carbamazepine dihydrate crystals as a function of agitation time

Figure S6 D-values of the broken carbamazepine dihydrate crystals as a function of agitation time at 1 mm and 120 RPM
